# Promoter hypermethylation and downregulation of trefoil factor 2 in human gastric cancer

**DOI:** 10.3892/ol.2014.1904

**Published:** 2014-02-21

**Authors:** PING JIANG, GUOYU YU, YONG ZHANG, YANG XIANG, ZHU ZHU, WEIYANG FENG, WENHUI LEE, YUN ZHANG

**Affiliations:** 1 Key Laboratory of Animal Models and Human Disease Mechanisms of the Chinese Academy of Sciences and Yunnan Province, Kunming Institute of Zoology, Chinese Academy of Sciences, Kunming, Yunnan 650223, P.R. China; 2 Department of Pathology and Pathophysiology, Kunming Medical University, Kunming, Yunnan 650500, P.R. China; 3 Department of Biochemistry, Kunming Medical University, Kunming, Yunnan 650500, P.R. China; 4 Department of Gastroenterology, The First Affiliated Hospital of Kunming Medical University, Kunming, Yunnan 650032, P.R. China

**Keywords:** trefoil factor 2, downregulation, gastric cancer, promoter hypermethylation

## Abstract

Trefoil factor 2 (TFF2) plays a protective role in gastric mucosa and may be involved in the progression of gastric cancer, but the detailed functions and underlying molecular mechanisms are not clear. The present study used a combination of clinical observations and molecular methods to investigate the correlation between abnormal expression of TFF2 and gastric cancer progression. TFF2 expression was evaluated by reverse transcription polymerase chain reaction (RT-PCR), quantitative PCR (qPCR), and western blot and immunohistochemistry analyses. TFF2 methylation levels were analyzed by genomic bisulfite sequencing method. The results showed that TFF2 mRNA and protein expression were decreased in gastric cancer tissues compared with the matched non-cancerous mucosa, and the decreased level was associated with the differentiation and invasion of gastric cancer. Moreover, the average TFF2 methylation level of CpG sites in the promoter region was 70.4% in three gastric cancer tissues, while the level in associated non-neoplastic tissues was 41.0%. Furthermore, the promoter hypermethylation of TFF2 was also found in gastric cancer cell lines, AGS and N87, and gene expression was significantly increased following treatment with a demethylating agent, 5-Aza-2′-deoxycytidine. In conclusion, TFF2 expression was markedly decreased in gastric cancer and promoter hypermethylation was found to regulate the downregulation of TFF2. TFF2 has been suggested as a tumor suppressor in gastric carcinogenesis and metastasis.

## Introduction

Gastric cancer is a multi-step progression from normal gastric mucosa to chronic gastritis, atrophy, intestinal metaplasia, dysplasia and ultimately cancer (1). Three closely related trefoil factors (TFFs) known in humans, pS2 (TFF1), spasmolytic polypeptide (SP or TFF2) and intestinal TFF (ITF or TFF3) (2,3), have been previously reported to be associated with the development of various types of cancer (4). TFF1, a tumor suppressor gene, exhibits decreased expression in precancerous and gastric cancer tissues (5,6). TFF3 expression is significantly elevated in intestinal metaplasia biopsy specimens compared with that in normal tissues, and the samples with an elevated expression of TFF3 lack goblet cell features (7). Furthermore, as TFF3 promotes tumorigenesis by increasing cell invasion and metastasis (8), gastric carcinoma patients with positive expression of TFF3 show invasive characteristics and poor prognosis (9). TFF2 is a principal cytoprotective TFF in the stomach and is highly expressed in ulcer tissue (10). Certain studies have previously reported that TFF2 expression is upregulated in gastric cancer tissues and that the overexpression is associated with cancer invasion, metastasis and a poor prognosis (11,12). However, several studies have shown that TFF2 expression is decreased significantly in gastric adenomas compared with the associated normal tissue, suggesting that the loss of TFF2 expression, as with the loss of TFF1, is an important event in gastric carcinogenesis (13–15). However, the correlation between the downregulation of TFF2 expression and clinicopathological data, as well as the detailed molecular mechanism underlying TFF2 abnormal expression, remain unclear.

The majority of gastric cancers are diagnosed in the advanced stage (16), which is generally resistant to radiotherapeutic or chemotherapeutic treatments. Therefore, it is important to identify any early regulatory molecules involved in gastric cancer progression, which may aid the detection of gastric cancer at an early and curable stage. Recently, it has been reported that the downregulated expression of protease-activated receptor 4 (PAR4) in gastric cancer tissues and the loss of PAR4 expression in gastric cancer may result from hypermethylation of the gene promoter (17). In the current study, we aimed to define the expression difference of TFF2 in gastric cancer and the gene methylation level.

## Materials and methods

### Gastric tissue samples

Gastric specimens (n=28) were obtained from the tumor and an adjacent non-cancerous area, ≥6 cm from the tumor tissues of gastric carcinoma patients at the First Affiliated Hospital of Kunming Medical College (Kunming, China). The mean age of the patients at diagnosis was 56 years. The non-neoplastic tissue was confirmed to lack tumor cell infiltration using histological analysis. The tissues were immediately placed in liquid nitrogen and stored at −80°C until use. A gastric cancer tissue microarray representing 110 types of gastric cancer with their non-neoplastic resection margins was constructed (18) at the Shanghai Outdo Biochip Center (Shanghai, China). Human samples were used in accordance with the requirements of the Ethical Committee of the Kunming Institute of Zoology, the Chinese Academy of Sciences, under the guidelines of the World Medical Assembly (Declaration of Helsinki). Written informed consent was obtained from the patient’s families.

### RNA extraction and polymerase chain reaction (PCR)

RNA extraction and first-strand cDNA synthesis were performed as previously described (19). For semi-quantitative reverse transcription PCR (RT-PCR) and quantitative PCR (qPCR), the following primers were used: Forward, 5′-CTGCTTCTCCAACTTCATCT-3′ and reverse, 5′-CTTAGTAATGGCAGTCTTCC-3′ for TFF2 (74-bp product); and forward, 5′-ATGGGGAAGGTGAAGGTCG-3′ and reverse, 5′-GGGGTCATTGATGGCAACAATA-3′ for glyceraldehyde 3-phosphate dehydrogenase (GAPDH; 107-bp product). GAPDH was used as an internal control. Following RT-PCR, the amplicons were separated by electrophoresis in a 2% agarose gel that was stained with ethidium bromide and viewed under ultraviolet illumination. qPCR was performed using a continuous fluorescence detector (Opticon Monitor; Bio-Rad, Hercules, CA, USA) and PCR was performed using an SYBR Green real-time PCR kit (Takara Bio, Inc., Dalian, China) with the following reaction conditions: Initial denaturation at 95°C for 1 min followed by 40 cycles at 95°C for 15 sec, 60°C for 15 sec and 72°C for 20 sec. Each sample was run three times. No-template controls (no cDNA in the PCR) were run to detect non-specific or genomic amplification and primer dimerization. Fluorescence curve analysis was performed using Opticon Monitor software. The relative quantitative evaluation of TFF2 levels was performed using the E-method (20) and expressed as a ratio of the TFF2 to GAPDH transcripts in the tumor tissue divided by that ratio in the non-neoplastic tissue of the same patient. The identities of RT-PCR and qPCR products were confirmed by DNA sequencing.

### Cell culture

AGS and N87 human gastric cancer cells were obtained from the American Type Culture Collection (Manassas, VA, USA). AGS cells were cultured in a 1:1 mixture of Dulbecco’s modified Eagle’s medium and Ham’s media. N87 cells were cultured in RPMI-1640 media containing 10% fetal calf serum, 100 U/ml penicillin and 100 mg/ml streptomycin. The cells were grown in a humidified atmosphere containing 5% CO_2_ at 37°C. The cells were seeded at a density of 1×10^6^ cells/ml in a 60 mm dish and treated with 10 mM 5-Aza-2′-deoxycytidine (5-Aza-2′-dC; Sigma-Aldrich, St. Louis, MO, USA). DMSO was used as a control. The cells were collected after 3 days and subjected to RT-PCR, qPCR and western blot analysis.

### Western blot analysis

Tissue and cell samples were homogenized in radioimmunoprecipitation assay buffer containing a protease inhibitor cocktail (Sigma-Aldrich). The protein concentration was determined using a protein assay kit (Bio-Rad). Samples (containing 50 μg of protein) were loaded into a sodium dodecyl sulfate-polyacrylamide gel electrophoresis gel, electrophoresed and then electro-transferred onto a polyvinylidene fluoride membrane. The membrane was subsequently blocked with 3% bovine serum albumin and incubated with an anti-human TFF2 polyclonal antibody (Protein Tech, Chicago, IL, USA) and a horseradish peroxidase-conjugated secondary antibody (Santa Cruz Biotechnology, Santa Cruz, CA, USA). Protein bands were visualized using Super Signal reagents (Thermo Fisher Scientific, Inc., Rockford, IL, USA).

### Tissue immunohistochemistry (IHC)

Tissue IHC was performed as previously described (21). Briefly, antigen retrieval was performed by heating samples in an autoclave at 121°C for 5 min. Dewaxed sections were pre-incubated with blocking serum and then incubated overnight with an anti-human TFF2 antibody (P-19; Santa Cruz Biotechnology) at 4°C. Specific binding was detected using a streptavidin-biotin-peroxidase assay kit (Maxim, Fujian, China). The section was counterstained with Harris hematoxylin. Direct microscopic micrographs were captured using a Leica DFC320 camera controlled using Leica IM50 software (Leica, Mannheim, Germany). Sections incubated with normal goat IgG served as negative controls, which were devoid of any detectable immunolabeling. The specificity of the anti-TFF2 antibody was confirmed using an overnight preincubation at 4°C with its antigen in a 20-fold molar excess of antigen to antibody. The preincubation with TFF2 antigens resulted in an absence of immunolabeling. Immunohistochemical staining was semi-quantitatively assessed by measuring the intensity of the staining (0, 1, 2 or 3) and the extent of staining (0, 0%; 1, 1–10%; 2, 11–50%; and 3, 51–100%). The scores for the intensity and extent of staining were multiplied to yield a weighted score for each case (maximum possible, 9). For the statistical analysis, the weighted scores were grouped into two categories, in which scores of 0–3 and 4–9 were considered negative and positive, respectively (22).

### Bisulfite sequencing

Genomic DNA from carefully selected 20-μm sections of gastric cancer, non-neoplastic tissues, and AGS and N87 cell lines was isolated using the Universal Genomic DNA Extraction kit (Takara, Bio, Inc.) and bisulfite-converted using the Clontech EpiXplore™ Methyl Detection kit (Takara, Bio, Inc.). TFF2 promoter sequences were amplified from the bisulfite-converted DNA by PCR, purified from agarose gels and subcloned into the pBackZero T Vector (Takara, Bio, Inc.). For each sample, 11 individual clones were sequenced to identify methylated cytosine residues. The PCR primer sequences used were forward, 5′-GGGATTTTTTTATGTTATTTGTTGG-3′ and reverse, 5′-ATAAAAAAACCCTCTCCTTCACTTACAAAA-3′.

### Statistical analysis

**Figure 1:**
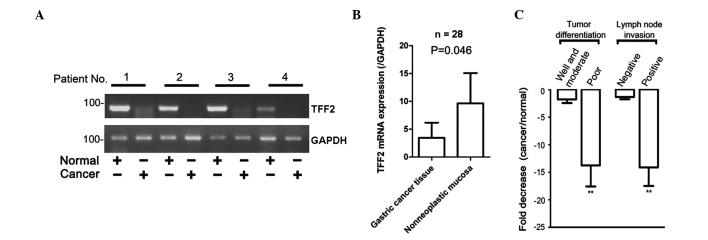
Downregulated expression of TFF2 mRNA in gastric cancer tissues and the correlation with clinicopathological data. (A) TFF2 mRNA expression in gastric cancer tissues was measured using RT-PCR. The matched normal and cancerous tissues from four patients, selected randomly, were analyzed using RT-PCR with TFF2- and GAPDH-specific primers (n=4). (B) TFF2/GAPDH mRNA expression levels in gastric cancer tissues were significantly decreased compared with those in the corresponding non-neoplastic mucosa tissues (P=0.046). (C) Correlation between TFF2 mRNA expression levels and tumor cell differentiation and lymph node metastasis in gastric cancer tissues. The expression of TFF2 was measured in the tissues of 28 gastric cancer patients using qPCR. TFF2 expression levels were compared between poorly differentiated and well- and moderately differentiated tumors. The levels among the tumors were also compared with lymph node metastasis and non-invasive tumors. Columns present the mean fold of decrease in the tumor tissues compared with that in non-neoplastic gastric tissues. ^**^P<0.05. TFF2, trefoil factor 2; RT-PCR, reverse transcription polymerase chain reaction; GAPDH, glyceraldehyde 3-phosphate dehydrogenase.

All statistical results were analyzed using SPSS 11.0 software (SPSS, Inc., Chicago, IL, USA). Fisher’s exact and χ^2^ tests were used to analyze the correlation between TFF2 expression and clinicopathological parameters (Tables I and II). Differences in the numerical data between the two paired groups were evaluated using the paired Wilcoxon test (Fig. 1). P<0.05 was considered to indicate a statistically significant difference.

## Results

### Downregulated expression of TFF2 mRNA in types of gastric cancer and correlation with clinicopathological parameters

TFF2 mRNA expression in gastric cancer tissues was examined using RT-PCR. In total, four pairs of samples were randomly selected from 28 patients and normalized to the GAPDH level. As shown in Fig. 1A, TFF2 mRNA expression was significantly decreased in cancer tissues compared with the associated normal mucosa. To quantify the differences in the expression of TFF2 mRNA, qPCR was performed on 28 gastric tumor tissue samples. TFF2 expression was downregulated in 93% (26 out of 28) of gastric cancer tissue samples compared with the associated non-neoplastic tissues. In addition, the mRNA levels of TFF2/GAPDH in gastric cancer tissues were significantly lower than those in the corresponding non-neoplastic mucosal tissues (mean ± SE, 3.4±2.7 vs. 9.6±5.4, respectively; P=0.046) (Fig. 1B). The clinical significance of the loss of TFF2 expression was further investigated based on the clinical pathological data. As shown in Table I, there were significant differences in TFF2 mRNA expression in well- and moderately differentiated tumors versus poorly differentiated tumors (P=0.019), and in tumors with lymph node invasion versus non-invasive tumors (P=0.026). In detail, TFF2 mRNA was reduced by a fold-change of 15.0±4.1 (mean ± SE) in the 22 poorly differentiated tumors compared with a fold-change of 1.9±0.7 in the six well- and moderately differentiated tumors (P=0.009; paired Wilcoxon test), and a fold-change of 15.6±3.7 in the 23 lymph node invasive tumors compared with a fold-change of 1.1 ± 0.4 in the five non-invasive tumors (P=0.002; paired Wilcoxon test) (Fig. 1C).

### Downregulation of TFF2 protein expression in gastric cancer tissues by western blot and tissue IHC analyses

**Figure 2:**
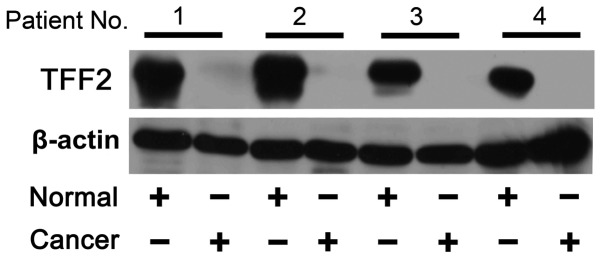
Western blot analysis of the downregulated expression of TFF2 protein in gastric cancer tissues. Western blot analysis tissue lysates from four cases of gastric cancer and relevant adjacent non-neoplastic mucosa. A significant loss of TFF2 was observed in the cancerous tissues compared with the matched normal tissues. The expression of β-actin served as a control. TFF2, trefoil factor 2.

**Figure 3:**
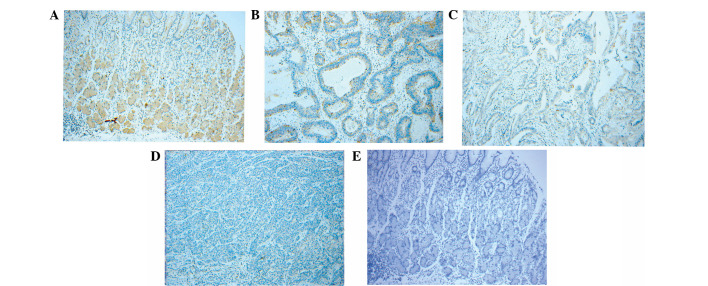
Immunohistochemical staining analysis of the downregulated expression of TFF2 protein in gastric cancer tissues and the correlation with clinicopathological data. Representative photomicrographs of immunohistochemical staining for TFF2 in paraffin-embedded sections of gastric tissues. (A) Normal stomach and (B) well-, (C) moderately and (D) poorly differentiated gastric cancer (intestinal type) tissues and (E) the absence of immunolabeling using anti-TFF2 antibody (P19) blocked with TFF2 antigen. Magnification, ×100. TFF2, trefoil factor 2.

The protein expression levels of TFF2 in normal and gastric cancer tissues were verified using western blot analysis. After the samples were normalized to the β-actin level, a marked reduction or loss of TFF2 protein was observed in four gastric tumor tissue samples compared with the matched non-malignant tissues (Fig. 2). TFF2 protein levels in normal and malignant gastric mucosa were also assessed using an IHC assay. In 110 gastric cancer tissue microarray assays, TFF2 expression was downregulated in 82% (90 out of 110). TFF2 was expressed at high levels in all investigated normal mucosa tissues and staining was identified from the basal-to-middle portions of gastric glands. Furthermore, TFF2 localization was in the cytoplasm and the membrane of gastric normal epithelial cells (Fig. 3A). However, the expression was significantly reduced in well- (Fig. 3B) and moderately (Fig. 3C) differentiated intestinal gastric cancer tissues, while TFF2 expression was almost absent in the poorly differentiated intestinal and diffuse types of gastric cancer (Fig. 3D). Sections incubated with normal goat IgG served as negative controls (Fig. 3E). Analysis of the correlation between TFF2 expression and clinicopathological data showed that decreased TFF2 expression was closely associated with tumor cell differentiation and lymph node invasion (Table II). In detail, TFF2 expression was decreased in 86.9% of poorly differentiated cancers and 65.4% of well- and moderately differentiated cancers (P=0.02; χ^2^ test). TFF2 expression was decreased in 88.8% of positive lymph node invasion and 63.3% of negative lymph node invasion tumors (P=0.004, χ^2^ test) (Table II).

### Treatment with 5-Aza-2′-dC increases TFF2 expression in the AGS gastric cancer cell line

**Figure 4:**
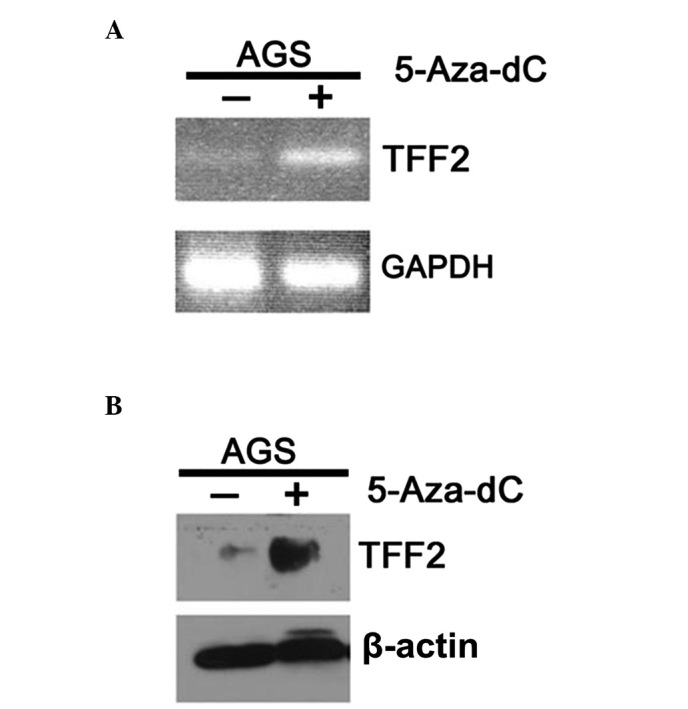
Treatment of 5-Aza-2′-dC restored TFF2 expression in AGS gastric cancer cells. The gastric cancer cell line, AGS, was incubated with 5-Aza-2′-dC (10 mM) or DMSO for 3 days and the expression level of TFF2 was assessed using (A) reverse transcription polymerase chain reaction and (B) western blot analysis. TFF2, trefoil factor 2; 5-Aza-2′-dC, 5-Aza-2′-deoxycytidine; GAPDH, glyceraldehyde 3-phosphate dehydrogenase.

To elucidate the potential molecular mechanisms underlying the process of TFF2 downregulation in the progression of gastric cancer, AGS cells were treated with 10 mM 5-Aza-2′-dC, which is a demethylating agent. RT-PCR analysis showed that TFF2 expression in AGS cells was significantly increased following 5-Aza-2′-dC treatment for 3 days (Fig. 4A). qPCR indicated a 3.89-fold increase in the mRNA expression levels of TFF2 following 5-Aza-2′-dC-treatment, while western blot analysis also indicated that TFF2 protein expression increased in AGS cells treated with 5-Aza-2′-dC (Fig. 4B). The results suggested that the epigenetic alteration may be involved in the downregulation of TFF2 expression in the progression of gastric cancer.

### Analysis of the promoter region methylation of the TFF2 gene in gastric cancer tissues

**Figure 5:**
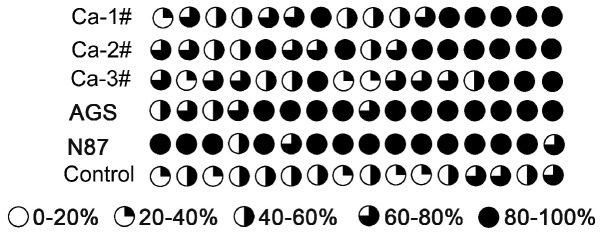
Genomic bisulfite sequencing of the TFF2 promoter-associated CpG sites in gastric cancer and non-neoplastic tissues. TFF2 promoter methylation in DNA from three gastric cancer tissues, one control of non-neoplastic tissue and two gastric cancer cell lines, AGS and N87. The average methylation at each analyzed CpG site in the TFF2 promoter was indicated based on the bisulfite sequencing of 11 individual clones. TFF2, trefoil factor 2.

Treatment with 5-Aza-2′-dC induced demethylation and led to the upregulated expression of TFF2 in AGS cells. Therefore, the methylation level of the TFF2 gene promoter was further analyzed in three gastric cancer and non-neoplastic tissue samples, as well as in AGS and N87 gastric cancer cell lines. Using the genomic bisulfite sequencing method, 16 CpG sites were analyzed in a 571-bp region containing part of the TFF2 promoter region. It included six CpGs found after the transcription start site and 10 CpGs located in the ~300-bp 5′-flanking region. The average promoter methylation level of three gastric cancer tissues was 70.4% and the control of non-neoplastic tissues was 41.0%, which showed that gastric cancer tissues with a decreased expression of TFF2 exhibited hypermethylation levels at the 16 CpG sites. In addition, AGS and N87 gastric cancer lines exhibited 85.2 and 93.7% methylation levels at the 16 CpG sites, respectively (Fig. 5). Therefore, these results indicated that promoter hypermethylation may lead to the inhibition of TFF2 transcription in gastric cancer.

## Discussion

TFFs are widely expressed in the mucosa of the gastrointestinal tract and play a role in inflammation, injury and repair. TFF2, a member of the TFFs, is expressed in the cytoplasm of gastric mucosal neck cells and acts as a mitogen to promote cell migration and suppress acid secretion (11). An SP-expressing metaplasia lineage is markedly associated with early gastric cancer and may be an important candidate for the development of metaplastic processes in gastric adenocarcinoma (23,24).

In the present study, by RT-PCR, qPCR, western blotting and immunohistochemical assays, the expression of TFF2 was shown to be frequently downregulated in gastric cancer tissues compared with the associated normal mucosa. In detail, TFF2 was expressed in the neck cells and the deeper glands of the normal gastric mucosa, but the expression was significantly decreased in the cancer tissues. Furthermore, no TFF2 expression was detectable in certain malignant tissues from poorly differentiated gastric cancer patients or highly lymph node-invasive cancer patients. The evidence that TFF2 expression was found to decrease is consistent with the results of previous studies, and decreased TFF2 expression is associated with the proliferation and malignant transformation of gastric cancer mucosa (15). However, the overexpression of TFF2 in gastric carcinoma tissues has also been shown in additional previous studies (12). The contradictory results may be attributed to the differences among cancer cell types. In the qPCR and IHC analyses of the current study, the decreased expression of TFF2 was 92.9% and 81.8%, respectively, and the reduced expression was found to significantly correlate with tumor cell differentiation and invasion. Therefore, there was reduced TFF2 expression in poorly differentiated tumor cells compared with well- and moderately differentiated tumor cells, and reduced TFF2 expression in positive lymph node invasion tumors compared with negative lymph node invasion tumors.

The dysregulation of TFF2 expression has been associated with gastric cancer cell migration, invasion and resistance to apoptosis. However, the underlying mechanisms associated with aberrant TFF2 expression remain unclear. Transcriptional silencing by promoter hypermethylation has emerged as one of the important mechanisms of gastric cancer development (25). TFF2 methylation has been shown to inversely correlate with mRNA levels of TFF2 at the time of *Helicobacter pylori* infection and to increase throughout gastric tumor progression (26). In the present study, a demethylating agent was found to increase the expression of TFF2 in AGS cells. Therefore, the methylation status of cytosines was further analyzed in sites of the TFF2 promoter region of gastric cancer and non-neoplastic tissues, as well as in AGS and N87 gastric cancer cell lines. Promoter hypermethylation was confirmed in gastric cancer tissues compared with that in non-neoplastic gastric mucosa. In addition, TFF2 promoter hypermethylation was also found in AGS and N87 gastric cancer cell lines. These results indicated that the TFF2 gene was undermethylated in the normal mucosa, but overmethylated in gastric cancer tissues, suggesting that promoter hypermethylation may lead to the inhibition of TFF2 transcription in gastric cancer tissues.

In conclusion, the current study showed that the expression levels of TFF2 were downregulated in gastric cancer tissues, particularly in poorly differentiated cancer cells and lymph node-positive tumors. Notably, the aberrant DNA promoter methylation is critical in the downregulation of TFF2 expression. These results may be useful to elucidate the molecular role of TFF2 in carcinogenesis and the progression and metastasis of gastric cancer.

## Figures and Tables

**Table I:** Correlation between TFF2 mRNA expression levels and clinicopathological data in gastric cancer patients.

		TFF2 mRNA levels				
			
		Not decreased (n=2)		Decreased (n=26)		
				
Clinicopathological parameters	Total (n=28)	n	%	n	%	P-value
Gender						
Male	18	0	0.0	18	100.0	0.119
Female	10	2	20.0	8	80.0	
Age, years						
≤65	17	1	5.9	16	94.1	1.000
>65	11	1	9.1	10	90.9	
Lauren type						
Intestinal	8	1	12.5	7	87.5	0.497
Diffuse	20	1	18.2	19	81.8	
TNM stages						
T1 and T2	7	2	0.0	5	100.0	0.056
T3 and T4	21	0	9.5	21	90.5	
Differentiation						
Poor	22	0	0.0	22	100.0	0.040
Well and moderate	6	2	33.3	4	66.6	
Lymph node metastasis						
Negative	5	2	40.0	3	60.0	0.026
Positive	23	0	0.0	23	100.0	

TFF2, trefoil factor 2.

**Table II:** Correlation between TFF2 protein expression levels and clinicopathological data in gastric cancer patients.

		TFF2 protein levels				
			
		Not decreased (n=20)		Decreased (n=90)		
				
Clinicopathological parameters	Total (n=110)	n	%	n	%	P-value
Gender						
Male	71	11	15.5	60	84.5	0.439
Female	39	9	23.1	30	76.9	
Age, years						
<65	63	15	23.8	48	76.2	0.086
≥65	47	5	10.6	42	89.4	
TNM stages						
T1 and T2	30	8	26.7	22	73.3	0.173
T3 and T4	80	12	15.0	68	85.0	
Lauren type						
Intestinal	81	17	21.0	64	79.0	0.268
Diffuse	29	3	10.3	26	89.7	
Differentiation						
Poor	84	11	13.1	73	86.9	0.020
Well and moderate	26	9	34.6	17	65.4	
Lymph node invasion						
Positive	80	9	11.3	71	88.8	0.004
Negative	30	11	36.7	19	63.3	

TFF2, trefoil factor 2.

## Abbreviations

TFFs: trefoil factor family
TFF2: trefoil factor 2
PCR: polymerase chain reaction
RT-PCR: reverse transcription PCR
IHC: immunohistochemistry
BSA: bovine serum albumin
GAPDH: glyceraldehyde 3-phosphate dehydrogenase
DMEM: Dulbecco’s modified Eagle’s medium
FCS: fetal calf serum
5-Aza-2′-dC: 5-Aza-2′-deoxycytidine
